# Meniscus root tears: state of the art

**DOI:** 10.1007/s00264-024-06092-w

**Published:** 2024-01-23

**Authors:** Ashraf T. Hantouly, Ghislain Aminake, Anfal Sher Khan, Muhammad Ayyan, Bruno Olory, Bashir Zikria, Khalid Al-Khelaifi

**Affiliations:** 1 Department of Orthopaedic Surgery, Surgical Specialty Center, Hamad Medical Corporation, Doha, Qatar; 2 Aspetar Orthopaedic and Sports Medicine Hospital, Doha, Qatar; 3 Weill Cornell Medicne, Cornell University, Doha, Qatar

**Keywords:** Knee, Meniscus root, Anterior root, Posterior root, Root repair

## Abstract

**Background:**

Meniscus root tears represent significant pathology that, historically, has been underdiagnosed and undertreated. However, the recognition of their clinical and functional significance has recently surged, mainly due to their frequent association with anterior cruciate ligament injuries.

**Aim:**

This comprehensive review discusses various aspects of meniscal root tears, including their epidemiology, biomechanics, etiology, clinical and radiological findings, classification, management and surgical techniques.

## Introduction

Meniscus root tears (MRTs) are characterized by radial tears within 1 cm of either the anterior or posterior meniscotibial attachment or by a complete avulsion of the meniscus from its tibial attachment [1, 2]. Historically, these injuries have been underdiagnosed and underappreciated, but they have gained substantial attention over recent years, primarily due to their frequent association with anterior cruciate ligament (ACL) injuries [3–5]. The first recorded meniscal root avulsion injury dates back to 1935 when Weaver, utilizing plain radiographs, described it as an ossification of the internal semilunar cartilage [6]. Subsequently, with the development of magnetic resonance imaging (MRI) in the early 1990s, Pagnani reported a case of medial meniscus subluxation associated with an avulsion injury to the posterior horn root attachment [7]. The modern definition of “meniscus root tear” was only introduced about a decade ago by Wolf Petersen and Robert Laprade [1, 5].

## Epidemiology

Meniscal root tears account for about 20% of all meniscal tears, representing a significant component within the spectrum of meniscus pathologies [3, 8]. Degenerative medial meniscus posterior root tears represent the most commonly reported subtype among meniscus tears. It is noteworthy that around 80% of all medial meniscus root tears were found in individuals who were obese with a sedentary lifestyle and those who were older than 50 years of age [1, 8]. Despite the contribution of these injuries to degenerative joint disease, they are often undiagnosed at initial presentation [2, 9].

Recent studies showed that the incidence of medial MRTs can be substantially higher in certain populations with distinctive lifestyles that entail frequent squatting, kneeling and sitting on the floor with folded legs [5]. On the other hand, lateral MRTs have been reported in 18% of chronic ACL-deficient knees and 12% of acute ACL injuries [2, 9]. With the recent growing interest in MRTs, it is plausible that the actual prevalence of these injuries is even higher than what is currently reported in the literature [1].

## Biomechanics

The menisci have a crucial role in knee function and biomechanics. They serve as shock absorbers by distributing the forces and converting the axial loads into radial tangential stresses, commonly referred to as hoop stresses [1, 2, 4]. Anchoring the meniscus to the tibia plateau and preventing meniscal extrusion during joint loading mitigate the impact of the compressive forces, consequently protecting the knee from potential osteoarthritic changes [10–13].

Several biomechanical studies have shown that meniscus root injuries are equivalent to total meniscectomy [12, 14]. These pathological alterations within the knee subsequent to a root tear are responsible for the gradual damage of the articular cartilage, leading to arthritis of the knee joint [15, 16].

In addition to their chondroprotective role, the menisci serve as secondary stabilizers of the knee joint [12, 17, 18]. Multiple factors, including the gross anatomy and histological composition of the meniscus, influence this stability. The orientation of fibers, along with the concavity of the superior surface, contributes to improved joint congruity, heightened load tolerance and multidirectional stability [19]. Additionally, several studies demonstrated the pivotal significance of the lateral meniscus posterior root in stabilizing the knee during both anterior tibial translation and pivoting activities [18, 20].

## Aetiology and mechanism of injury

Posterior roots of the menisci are more commonly affected than the anterior ones, which can be iatrogenically injured during ACL tibial tunnel drilling and intramedullary nailing of tibial shaft fractures [20–22]. Medial MRTs have been commonly, but not only reported in older patients, females, obese patients with a sedentary lifestyle. These tears are closely correlated with age-related degenerative changes and low-energy repetitive trauma such as deep squatting, high knee flexion postures and descending knee motion [18, 20, 23]. In contrast, lateral MRTs are often found in young male patients with acute ACL or multiple ligamentous knee injuries [1, 3, 4, 20] (Table 1). Studies have a significantly higher concomitance between ACL injuries and lateral MRTs compared to medial MRTs, with the risk being elevated tenfold [24].

**Table 1:** Risk factors for meniscus root tear

Medial meniscus root tear	Lateral meniscus root tear
Female	Male
Obesity	Multi ligament injury
Older age > 50	Chronic ACL deficient knee
Sedentary lifestyle	Physically active lifestyle
Frequent squatting and kneeling	
Middle East and Asian populations	

## Clinical features

Diagnosing MRTs can be a complex and challenging task due to the limited sensitivity and specificity of the associated signs and symptoms [1, 2]. Hence, it is imperative to stress on the importance of comprehensive history taking, meticulous physical examination and appropriate imaging techniques to ensure timely and accurate diagnosis.

### Clinical presentation

Root tears are not confined to acute knee trauma and are often correlated with a chronic degenerative process. Hence, it is imperative to maintain a high degree of suspicion when assessing patients who exhibit risk factors such as advanced age, increased activity level, sedentary lifestyle and specific injury mechanisms [1, 9].

Older patients with MRTs tend to present with chronic and subtle symptoms frequently associated with repetitive low-energy mechanisms, such as deep squatting and kneeling on the floor [1, 20]. In contrast, among younger patients, such injuries often result from a rotatory blow to a flexed knee or as part of a multi-ligamentous knee injury [20].

Patients typically present with posterior knee pain and restricted range of motion, particularly during flexion. Additionally, patients might describe mechanical symptoms, such as locking and clicking or episodes of instability [3, 4].

### Physical examination

During physical examination, patients frequently exhibit tenderness along the joint line and decreased flexion angle [25]. In cases of medial meniscus posterior root tears, an extruded meniscus can be palpated. This can be provoked by applying a varus stress force while the knee is in full extension [26]. Additionally, McMurray and Thessaly tests might be positive. Given the potential concomitance with other knee injuries, it is imperative to always conduct a comprehensive knee examination, especially with high-grade pivot shift and anterior drawer tests [8, 27].

## Radiological findings

The growing interest and recognition of the clinical impact of MRTs have increased the rate of their radiographic diagnosis and detection [1]. Plain X-rays, including anteroposterior, lateral, weight-bearing 45° posteroanterior and Merchant views, are typically requested to assess and rule out bony abnormalities. Additionally, cone-bean weight-bearing CT can be used to assess for meniscus extrusion [28]. However, MRI is the imaging modality of choice to diagnose meniscal root tears and concomitant pathologies [20, 29]. MRI has higher sensitivity but less specificity in detecting medial meniscus posterior root tears as compared to lateral meniscus root tears [29]. This discrepancy in sensitivity can be attributed to the anterior oblique course of the lateral meniscus posterior root attachment and its proximity to other structures [1, 29, 30].

The quality of the MRI and the skill level of the radiologist may affect the ability to detect meniscus root tears on MRI. Nevertheless, diagnosis can be guided by the direct and indirect signs of meniscus root tears, which are listed in Table 2.

**Table 2:** Signs of meniscus root tear on MRI

	Direct signs	Indirect signs
T2 sagittal view	Ghost sign	Subchondral bone oedema
T2 coronal view	Cleft/truncation sign	Meniscus extrusion of more than > 3 mm
T2 axial view	Increased signal at the root area/radial tear	Parameniscal cyst

**Fig. 1:**
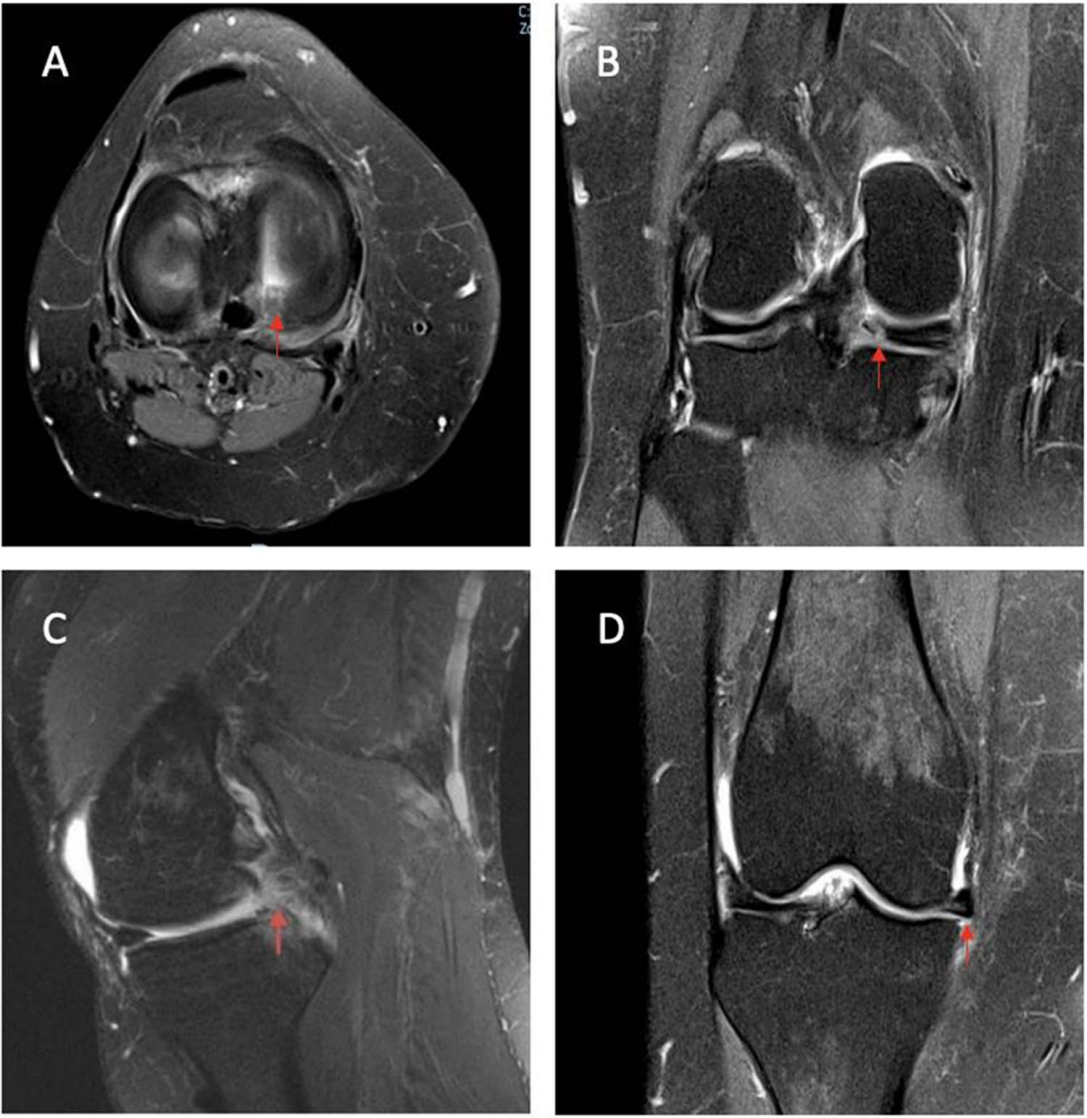
Direct and indirect MRI signs of meniscal root tears. T2 MRI images showing **A** high signal in the region of meniscus root and posterior horn on axial images; **B** Cleft sign; **C** Ghost sign and subchondral edema on sagittal image; and **D** meniscus extrusion on coronal image

**Fig. 2:**
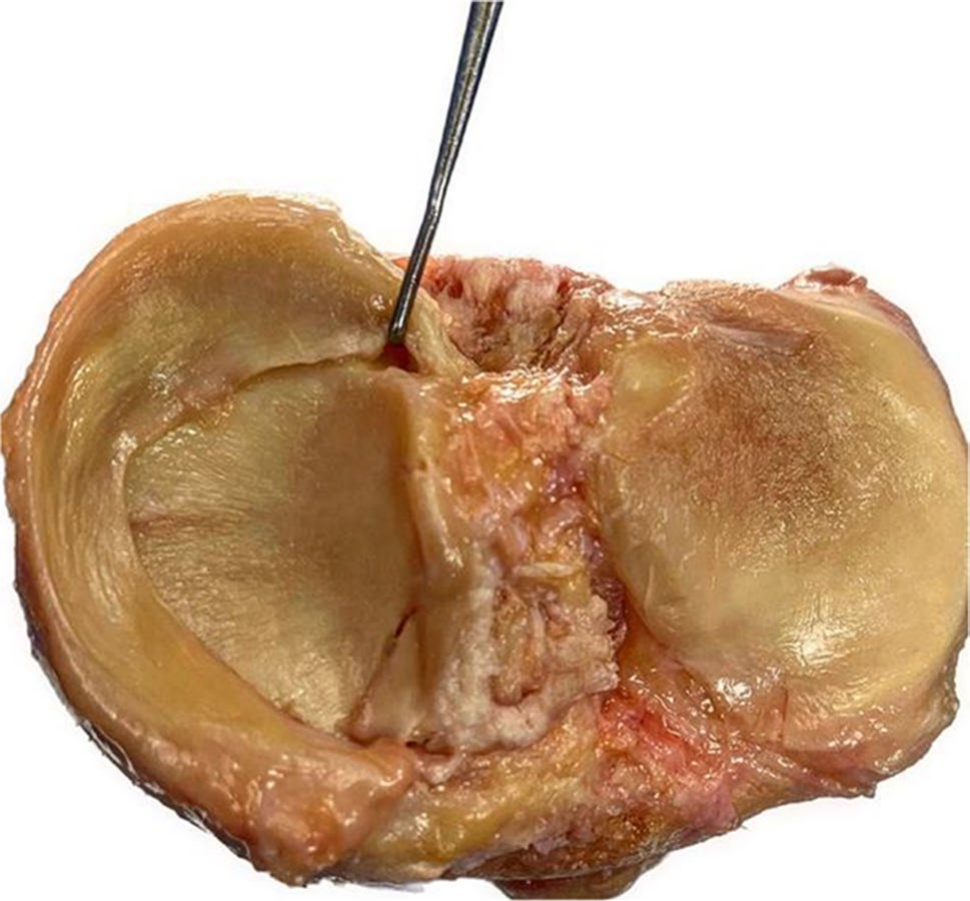
Cadaveric photograph of the knee showing the medical meniscus and its posterior root

**Fig. 3:**
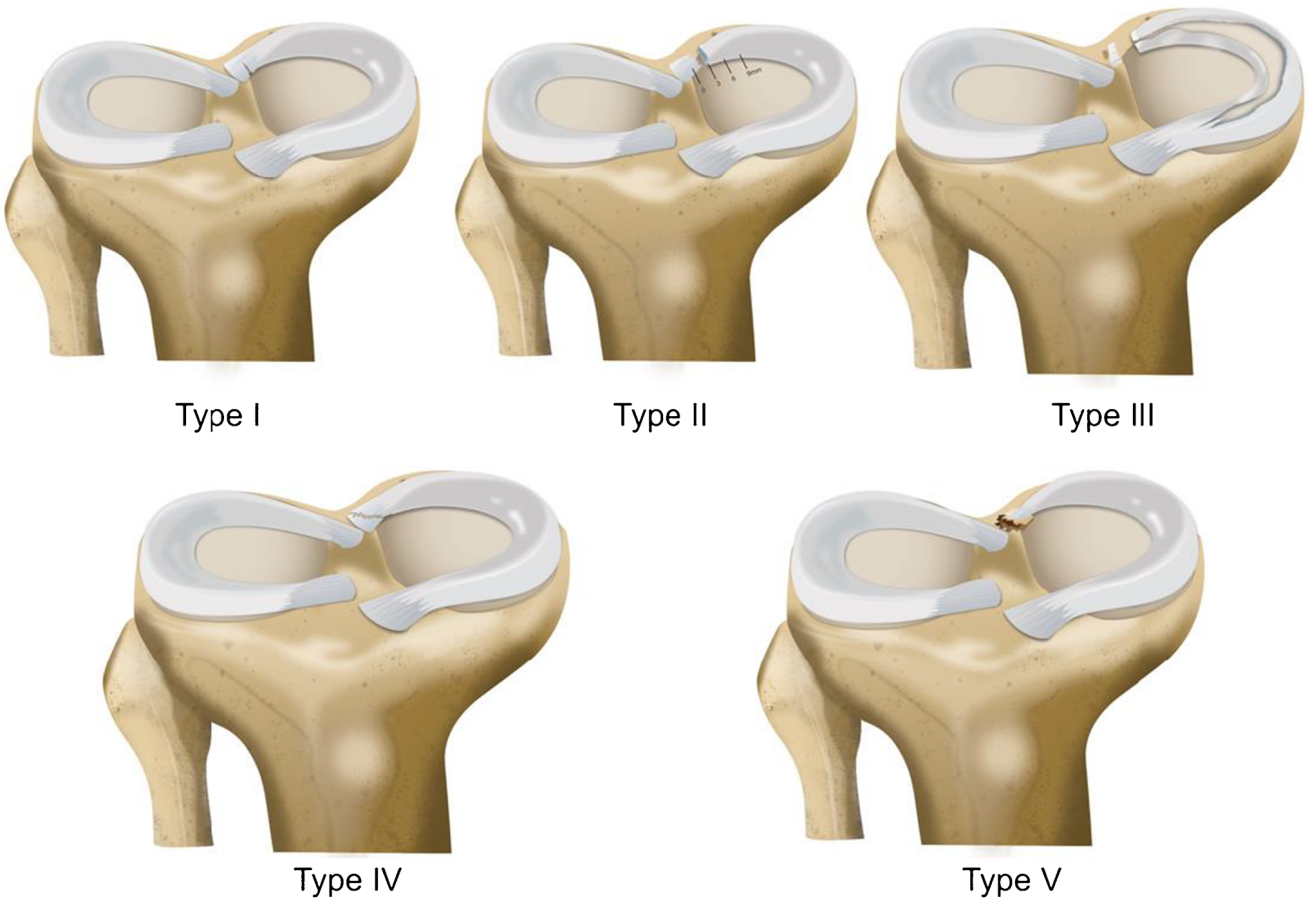
Laprade’s arthroscopic morphological meniscal root classification system [34]. Type 1: Stable partial tear. Type 2A: Full tear within 3 mm of root attachment. Type 2B: Full tear within 3–6 mm of root attachment. Type 2B: Full tear within 6–9 mm of root attachment. Type 3: Full tear with bucket handle tear. Type 4: Complex oblique full tear. Type 5: Root bony avulsion [34]

The direct signs of meniscal root tears on MRI include (1) high signal in the region of the meniscus root and posterior horn on axial images (Fig. 1A), (2) “Cleft” sign, representing a vertical linear tear on the meniscal root on coronal T2 MRI (Fig. 1B), and (3) “Ghost” sign, representing the absence of a normal meniscal signal on sagittal T2 MRI (Figure 1C). Indirect signs include parameniscal cysts, subchondral bone marrow edema and meniscal extrusion >3 mm (Fig. 1D) [23, 29, 31].

Despite the high sensitivity and specificity of MRI in diagnosing MRTs, it is essential to acknowledge that some of these tears may remain undetected until an arthroscopic procedure is performed. Hence, arthroscopic visualization remains the gold standard for diagnosing meniscal root tears [20, 32–34].

## Classification

Several classification systems have been proposed to describe and guide the management of MRTs. However, Laprade’s classification is considered the most commonly used one [34]. Laprade classified MRTs based on their morphology during arthroscopic assessment. The classification encompasses five distinct lesions: type 1 tears representing partial root tears that are stable (7% of all meniscus root tears). Type 2 tears representing complete radial tears within 9 mm of root attachment center (67.6%). This type was subclassified into three subtypes according to the distance of the tear from the center of the root attachment (2A 0–3 mm, 2B 3–6 mm and 2C 6–9 mm). Type 3 tear was defined as bucket-handle tear with complete root detachment (5.6%). Type 4 tears are complex oblique tears with complete root detachment (10%) and bony avulsion of the root attachment (9.9%) [34]. Figures 2 and 3 demonstrate a cadaveric photograph of the knee showing the medical meniscus and Laprade’s arthroscopic classification of meniscal root tears, respectively.

## Treatment algorithm

The management of MRTs depends on several factors, including injury severity, time of injury and articular cartilage status [12, 33]. Given the crucial role of the menisci in articular cartilage preservation, saving the meniscus should always be a priority. Meniscal root tears have three main treatment options: conservative treatment, partial meniscectomy and surgical repair [32, 33].

Non-operative management is indicated in certain situations including cases of advanced articular cartilage damage and for older or obese patients who are not medically fit for surgery [18, 20, 32]. This approach typically includes symptomatic treatment with pain medications, physical therapy, lifestyle modification and an unloader brace [20, 32].

**Fig. 4:**
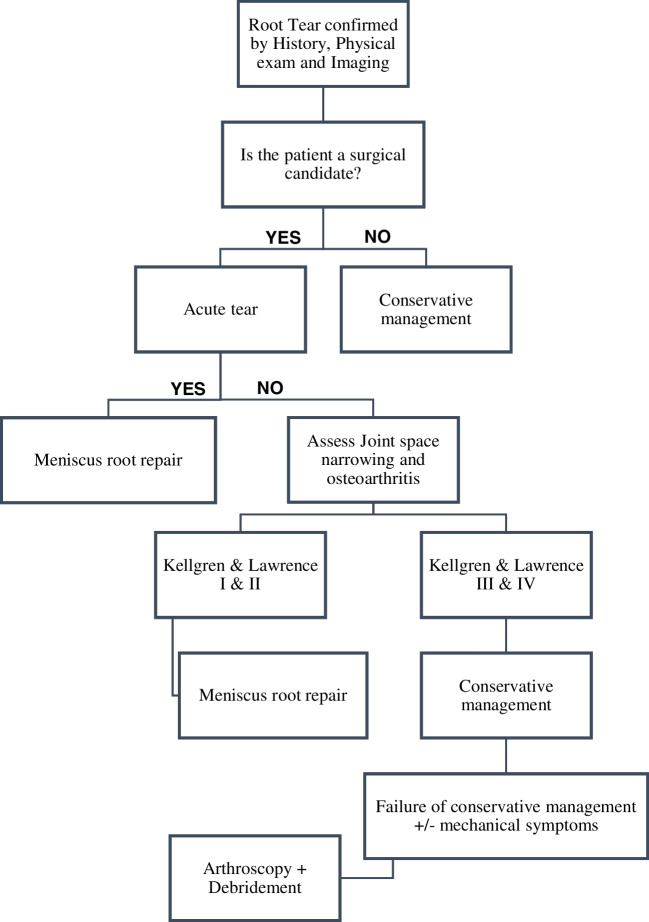
Meniscus root tears treatment algorithm

The mainstay of MRTs treatment is surgical repair with the primary objective of restoring knee kinematics by preserving the meniscal function in order to delay the onset of osteoarthritis [35]. However, advanced osteoarthritis, focal chondral injury, severe coronal and sagittal malalignment and knee instability are all considered contraindications to meniscal root repair [9, 20, 30, 31]. Therefore, addressing these pathologies is imperative before considering surgical repair. For patients with persistent mechanical symptoms despite non-operative measures, partial meniscectomy may be indicated if repair is deemed unfeasible. While partial meniscectomy offers short-term relief, it is crucial to acknowledge that the long-term consequence invariably involves the development of further osteoarthritis [15]. Figure 4 illustrates the treatment algorithm for MRTs.

## Surgical technique

### Arthroscopic posterior meniscus root repair

Given the essential role of the meniscus root in the proper function of the meniscus, immediate surgical intervention is strongly recommended for root injuries in young and active patients with minimal articular cartilage injury [36, 37]. Several fixation techniques have been described in the literature, yet the two main techniques used to fix the posterior root of the meniscus are suture anchor and transtibial pullout techniques. The transtibial pullout repair technique is considered the gold standard method as it provides a more anatomical reduction, which is crucial to restore meniscus stability and knee kinematics [18, 20, 37–39].

**Fig. 5:**
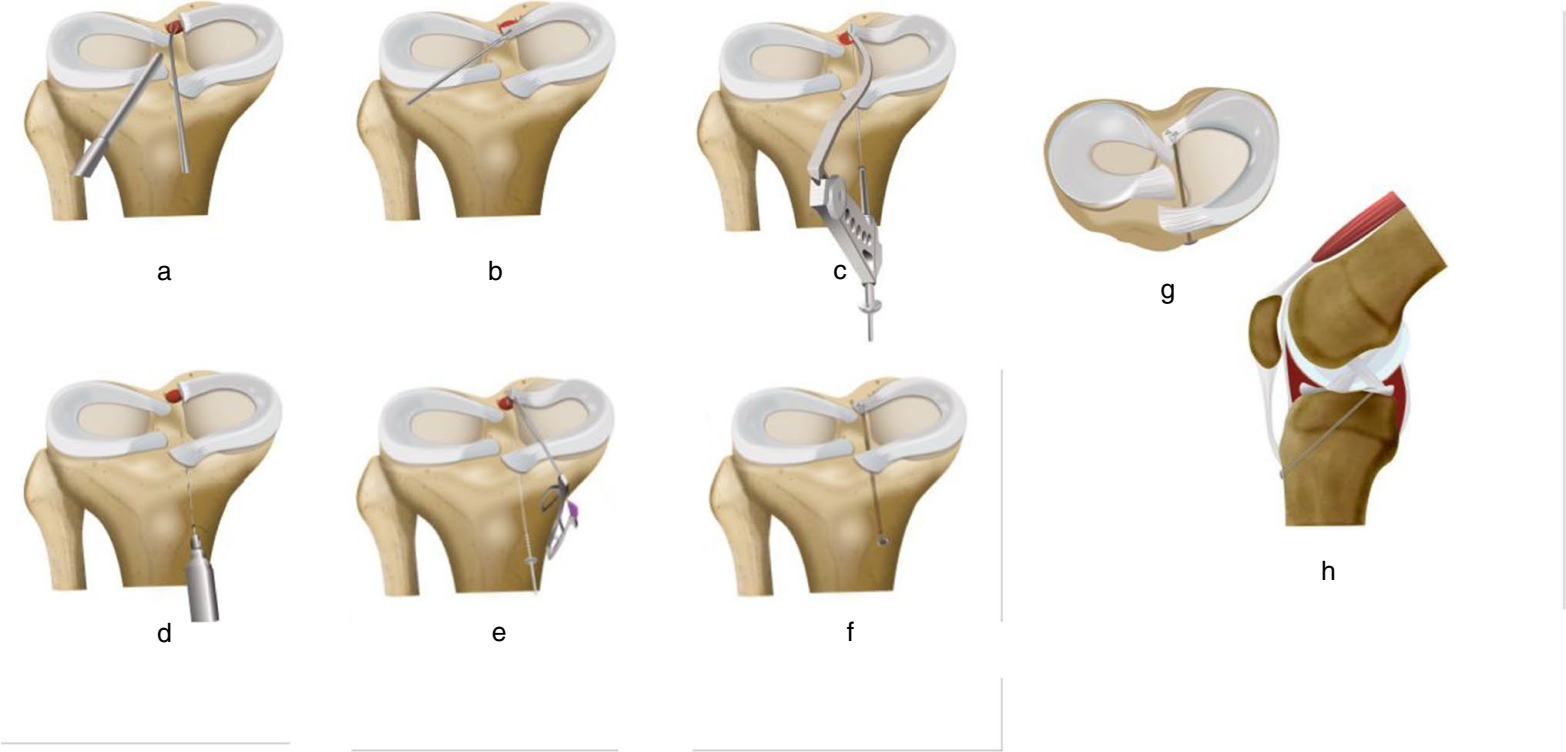
Single tunnel transtibial fixation of medial meniscal root tear. **a** Preparation of the root attachment site by promoting bleeding at the root bed using a curette. **b** Pulling the root to its anatomic attachment site. **c**, **d** Drilling an ipsilateral single transtibial tunnel using meniscal root or ACL guide. **e** Two luggage tag sutures or looped sutures are placed in the detached root and then retrieved using a suture passer. **f** Sutures are secured on the anterior aspect of the tibia using a button. **g**, **h** Axial and sagittal views of the final fixation, respectively

**Fig. 6:**
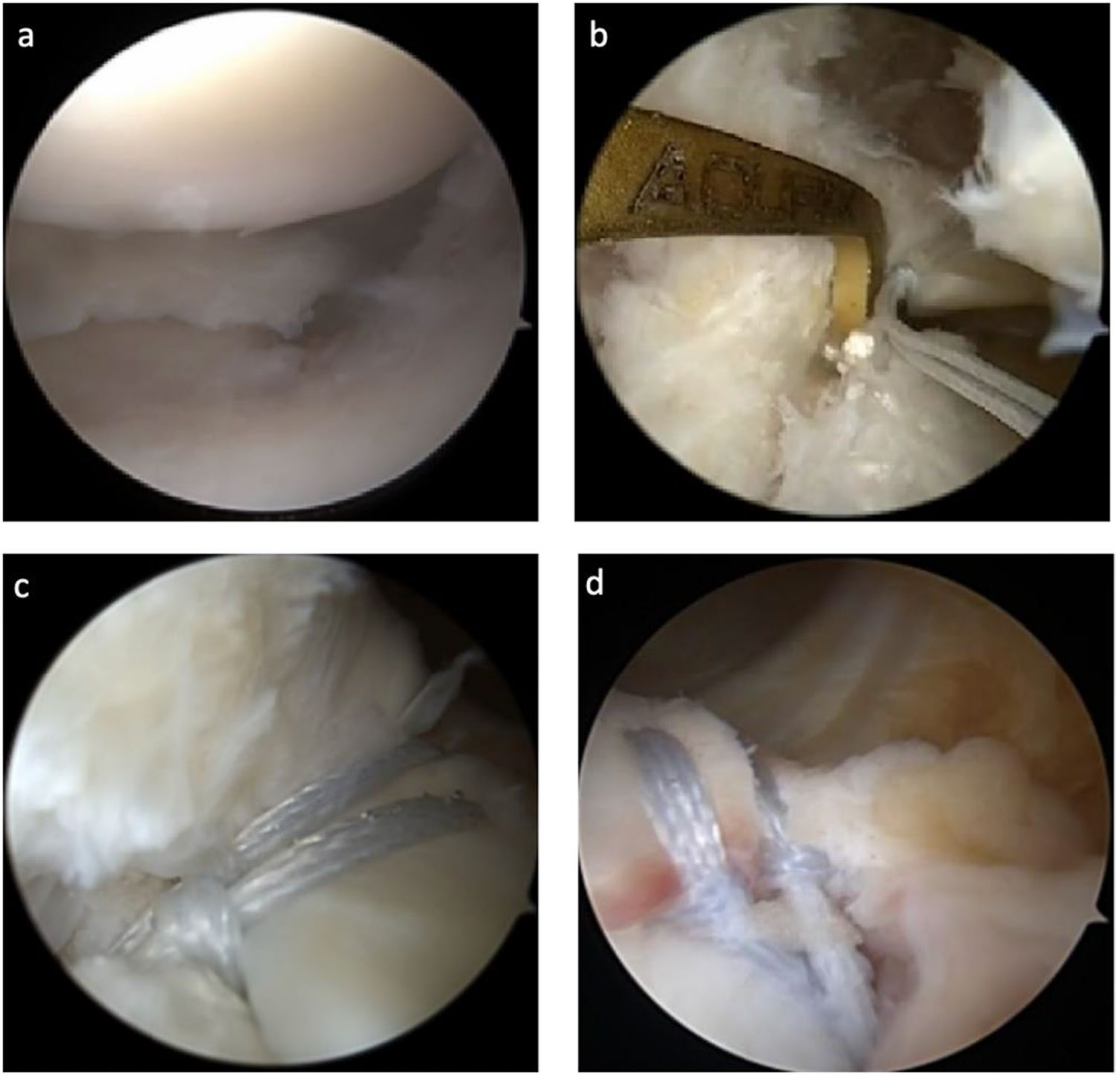
Arthroscopic images of a tear of the posterior horn of the medial meniscus (PHMM) and its repair. **a** Tear of the posterior horn of the medial meniscus. **b** The guide is placed in the anatomical position of the PHMM to steer the transtibial drilling. **c**, **d** Two luggage tag sutures are passed through the meniscal root under arthroscopic visualization and then passed through the transtibial tunnel

The transtibial pullout repair consists of drilling a tunnel, steered by the meniscal root guide, through the anterior aspect of the ipsilateral proximal tibia, extending to the anatomic root insertion site. Luggage tag or looped sutures are passed through the meniscal root under arthroscopic visualization. Subsequently, these meniscal sutures are retrieved through the tunnel and firmly secured over a cortical fixation device such as a button or an anchor [20, 30] (Fig. 5 and Fig. 6).

### Centralization

**Fig. 7:**
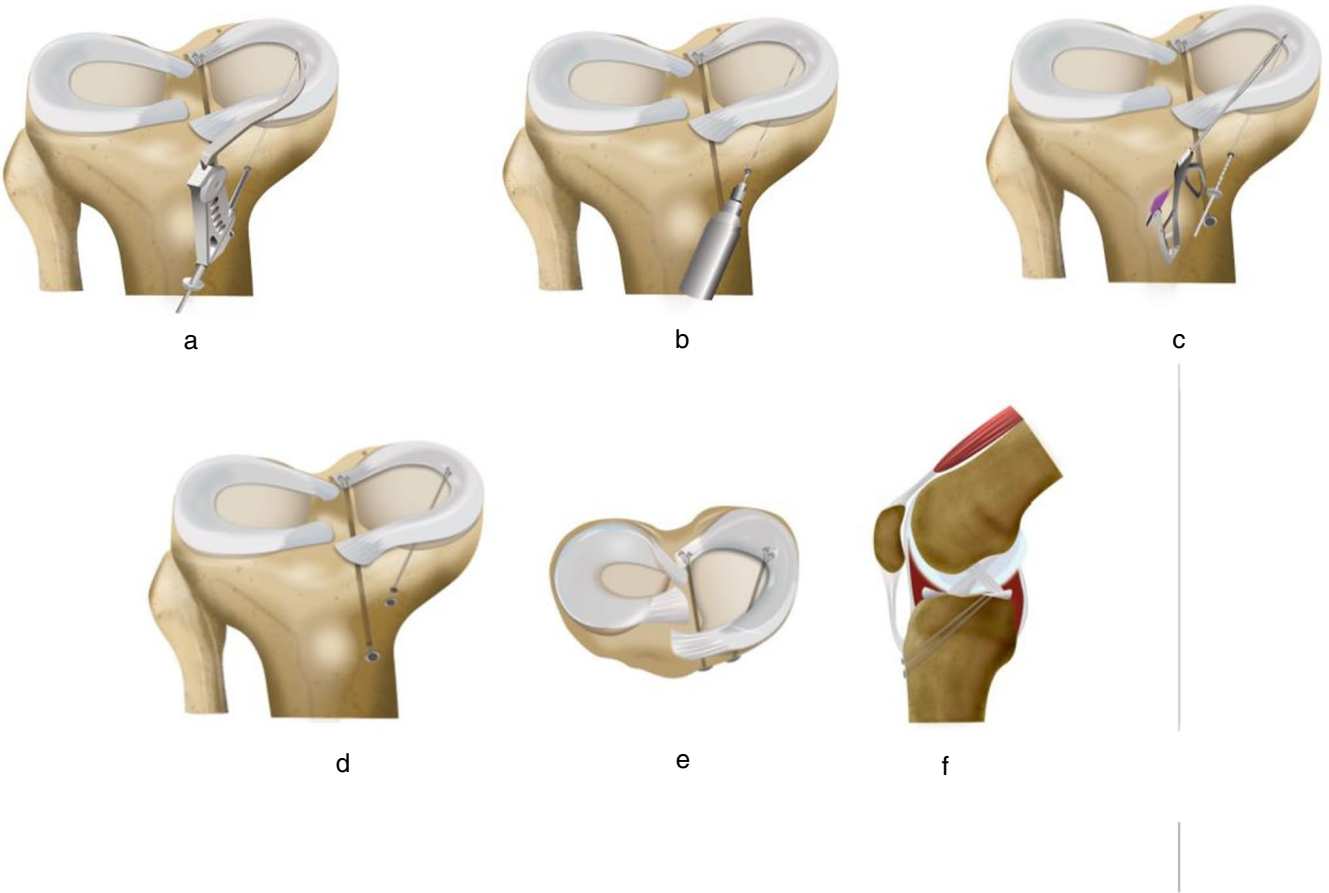
Single tunnel transtibial centralization of the medial meniscus. **a**, **b** Drilling an ipsilateral single transtibial tunnel using meniscal root or ACL guide. **c** Two luggage tag sutures are placed in the body of the meniscus and then retrieved using a suture passer. **d** Sutures are secured on the anterior aspect of the tibia using a button. **e**, **f** Axial and sagittal views of the final fixation, respectively

To enhance meniscus root repair, centralization of the extruded meniscus can be employed, as extrusion commonly arises as a complication of meniscus root repair. Meniscus centralization is performed by reducing and fixing the extruded meniscus to the articular surface of the tibia at the meniscocapsular junction (Fig. 7).

### Suture anchor

The suture anchor technique is typically performed via a posteromedial or lateral portal, with the occasional use of an accessory posterior portal for suture passing. Ensuring optimal outcomes necessitates adequate visualization and careful consideration of the trajectory of the native posterior root attachment.

## Post-operative rehabilitation

The existing evidence regarding rehabilitation after MRTs repairs is primarily derived from cadaveric and animal studies, as comparative studies between different protocols are lacking [24]. However, most rehabilitation protocols in the literature follow four common stages [37, 40].

Phase 1 (0–6 weeks) consists of protecting the repair, resolving joint inflammation, swelling and regaining leg control. During this phase, the patient is maintained non-weight bearing for at least six weeks with a hinged knee brace locked in extension. Scheduled passive range of motion (ROM) exercises are arranged with a physiotherapist, and the range of motion restricted is to 0–90° of flexion to avoid deep flexion as it is associated with increased peak contact pressure and posterior extrusion forces on the meniscus.

The second phase (6–12 weeks) focuses on gaining full weight bearing, better leg control and normal gait without crutches. At the beginning of this phase (6–8 weeks), passive ROM is maintained up to 90° and active ROM is gradually initiated but limited to 0–60° of flexion. Weight-bearing knee flexion is introduced after the patient has regained normal controlled gait and is limited to 45° of flexion. Passive ROM can be increased gradually beyond 90° at eight weeks. At the end of this stage, the patient should be able to perform daily live activities without a brace or crutches comfortably.

The third phase (12–18 weeks) consists of maintaining ROM, proprioception and strengthening. During this phase, the patient is introduced to sports and work-specific activities. Care should be taken to avoid significant swelling after exercising. Weightbearing high flexion or deep squats above 70° are prohibited at this stage.

The last phase (above 21 weeks) focuses mostly on sport/activity-related exercises in preparation for a return to sport for athletes, strengthening and full body fitness including cardiovascular exercises. Care should be taken to avoid severe pain during the exercises and significant swelling following the exercises.

## Prognosis

Although meniscal root repair aims to restore the meniscus root anatomy, biomechanical studies have demonstrated that such repairs only partially reinstate the knee’s native kinematics. Consequently, despite the advancements in root repairs, they fall short of completely preventing the progression of knee osteoarthritis [15, 41]. However, patients who underwent anatomic meniscal root repair have demonstrated significant improvement with regard to pain, function and level of activity [42]. Furthermore, these repairs have been correlated with a slower progression of osteoarthritis [42].

## Data Availability

Not applicable.
